# First-Principles Calculation of the Third Virial Coefficient of Helium

**DOI:** 10.6028/jres.114.018

**Published:** 2009-10-01

**Authors:** Giovanni Garberoglio, Allan H. Harvey

**Affiliations:** CNISM and Dipartimento di Fisica, Università di Trento, via Sommarive 14, 38100 Povo (TN), Italy; Thermophysical Properties Division, National Institute of Standards and Technology, 325 Broadway, Boulder CO 80305, USA

**Keywords:** calibration, density, helium, metrology, path integral, thermodynamic properties, virial coefficients

## Abstract

Knowledge of the pair and three-body potential-energy surfaces of helium is now sufficient to allow calculation of the third density virial coefficient, *C*(*T*), with significantly smaller uncertainty than that of existing experimental data. In this work, we employ the best available pair and three-body potentials for helium and calculate *C*(*T*) with path-integral Monte Carlo (PIMC) calculations supplemented by semiclassical calculations. The values of *C*(*T*) presented extend from 24.5561 K to 10 000 K. In the important metrological range of temperatures near 273.16 K, our uncertainties are smaller than the best experimental results by approximately an order of magnitude, and the reduction in uncertainty at other temperatures is at least as great. For convenience in calculation of *C*(*T*) and its derivatives, a simple correlating equation is presented.

## 1. Introduction

Accurate knowledge of the thermophysical properties of helium is desirable for many applications in metrology. Examples include microwave resonance measurements for development of a pressure standard [1], measuring the Boltzmann constant [2], and acoustic gas thermometry [3].

In many cases, the nonideal behavior of the gas is a major component of the overall uncertainty. That non-ideality is expressed in the virial expansion

$$\frac{p}{\rho RT}=1+B(T)\;\rho +C(T)\;\rho^{2}+\ldots ,$$ (1)

where *p* is the pressure, *ρ* the molar density, *R* the molar gas constant, and *T* the absolute temperature. *B*(*T*) is the second virial coefficient, representing the lowest-order deviation from ideal-gas behavior. As the density increases, the contribution from the third virial coefficient *C*(*T*) becomes significant. *B*(*T*) is a function only of the interactions between pairs of molecules, while *C*(*T*) is determined by interactions among three molecules.

Because the helium atom has only two electrons, and because of advances in methodology, algorithms, and computing power for *ab initio* quantum calculations, it is now possible to calculate properties of individual helium atoms and pairs of atoms with very high accuracy. The use of such calculated values to develop standards for thermophysical properties was first proposed by Aziz *et al.* [4], after which Hurly and Moldover [5] calculated helium’s second virial coefficient *B*(*T*), dilute-gas viscosity, and dilute-gas thermal conductivity with uncertainties smaller than those of the best experiments. Hurly and Mehl [6] recently improved on this work, primarily by using a more accurate pair potential. Bich *et al.* [7] performed similar calculations of pair quantities with another high-accuracy pair potential [8]. They also calculated *C*(*T*) with quantum effects considered at a first-order perturbation level, but (as explained in Sec. 2.2) the Axilrod-Teller term they used for the three-body potential is not a good representation of three-body effects for helium.

For the third virial coefficient *C*(*T*), rigorous first-principles calculations are more difficult. While classical calculation of *C*(*T*) for spherically symmetric species is straightforward [9], quantum effects must be considered for a light gas such as helium. Semiclassical perturbation approaches have been derived [10, 11], but they will be insufficient at low temperatures and their uncertainty is difficult to quantify. What is needed is a fully quantum approach as is available for *B*(*T*) [9], but this problem has not been solved in closed form for *C*(*T*).

Early quantum calculations, aimed to elucidate how *C*(*T*) depends on the inter-molecular potentials, were performed at the beginning of the 1960s, using the general theoretical framework developed by Lee and Yang [12]. Pais and Uhlenbeck [13] as well as Larsen [14] discussed various approximations to the full quantum problem, and these results were used to estimate the binding energy. Other calculation attempts tried to imitate the exact quantum calculations of *B*(*T*) based on scattering phase shifts. Larsen and Mascheroni [15] were able to obtain a rigorous expression for the third virial coefficient under the (unrealistic) assumption of the absence of bound states. Reiner [16] developed a calculation method based on the Faddeev equations for the quantum-mechanical three-body problem, but the difficulties in solving the coupled integral equations could not be overcome, and approximations were required.

The first rigorous quantum calculation of virial coefficients was developed by Fosdick and Jordan [17–19] who derived a path-integral expression for the third virial coefficient in the presence of two-body pairwise additive forces, and showed in detail how to evaluate it numerically in the case of a simple Lennard-Jones model for He.

Fosdick and Jordan’s approach was independently rediscovered by Diep and Johnson [20] who devised, by analogy to the classical expression, a path-integral formula for the second virial coefficient of a quantum gas. They used their expression to calculate the second virial coefficient for a new H_2_-H_2_ potential that they had computed using *ab initio* calculations, neglecting the rotational degrees of freedom. Their expression was generalized to the case of asymmetric rotors by Schenter [21] who used it to calculate the second virial coefficient of a model of water.

Recently, we developed a rigorous path-integral Monte Carlo procedure to calculate the second virial coefficient of molecular hydrogen, extending the approach pioneered by Fosdick and Jordan [17] to take into account the rotational degrees of freedom of linear molecules. We used this method together with state-of-the-art *ab initio* calculations of the pair potential to obtain good agreement with experimental data in a wide range of temperatures [22].

In the present work, we further extend the path-integral approach employed by Jordan and Fosdick for spherical particles [18] to calculate the third virial coefficient of ^4^He in the presence of nonadditive three-body interactions. We use this method to calculate the third virial coefficient of ^4^He from 24.5561 K to 10 000 K, using a recent *ab initio* derived three-body potential [23].

## 2. Intermolecular Potentials

### 2.1 Pair Potential

We write the pair potential as *U*_2_(*r*), where *r* is the center-to-center distance between the atoms. We use the pair potential known as *ϕ*_07_, which was developed by Hurly and Mehl [6] based on the best *ab initio* calculations available in 2007. For uncertainty analysis, we also use their potentials $\phi_{07}^{-}$ and $\phi_{07}^{+}$, which represent uncertainty limits (expanded uncertainty with coverage factor *k* = 2) for the *ϕ*_07_ potential.

While this work was in progress, a pair potential of higher accuracy (consistent within mutual uncertainties with *ϕ*_07_) was published by Jeziorska *et al.* [24]. Because our uncertainties are dominated by the three-body potential (see Sec. 4.2), our results would not have been significantly different had we used that pair potential.

### 2.2 Three-Body Potential

Calculation of *C*(*T*) also requires knowledge of the nonadditive three-body contribution to the potential energy in a triplet of atoms. We write this as *U*_3_(*r*_12_, *r*_13_, *r*_23_), where *r_ij_* is the distance between atoms *i* and *j*. The three-body potential of helium has been studied by Cencek *et al.* [23], who developed separate *ab initio* potentials based on symmetry-adapted perturbation theory (SAPT) and coupled-cluster (CC) calculations. The two potentials were estimated to have a maximum uncertainty of 10 %, but very recent work [25] has shown that the SAPT potential is significantly less accurate, while the CC potential is in good agreement (better than 10 %) with higher-level calculations. We therefore employ the CC potential in this work. For purposes of uncertainty analysis, we interpret the estimated 10 % uncertainty as an expanded uncertainty at the *k* = 2 level. In analogy to the procedure of Hurly and Mehl [6] for the pair potential, we define for our uncertainty analysis three-body potentials CC− (obtained by multiplying the potential by 1.1 where it is negative and by 0.9 where it is positive) and CC+ (multiplying by 0.9 where it is negative and by 1.1 where it is positive).

It is worth noting that, at most temperatures of interest, the main three-body effect for helium is the non-additivity in repulsive forces. The commonly used Axilrod-Teller three-body term [26] accounts only for dispersion interactions. At all but the lowest temperatures considered in this work, the reduced temperature is quite high (compared to the characteristic temperature for the pair dispersion interaction of approximately 11 K), and three-body dispersion effects are less important than three-body repulsion effects. Therefore, an Axilrod-Teller term would seriously misrepresent the three-body potential, even giving a contribution to *C*(*T*) of the wrong sign at moderate and high temperatures.

## 3. Calculation Methods

Let us denote by *Q_N_*(*V,T*) the partition function of *N* particles in a volume *V* at a temperature *T*. By defining the quantities *Z_N_* as

$$\frac{Z_{N}}{N!}\equiv \frac{Q_{N}(V,T)V^{N}}{Q_{1}{\left(V,T\right)}^{N}},$$ (2)

then the expressions for the second and third virial coefficients, *B*(*T*) and *C*(*T*), become [9]

$$B(T)=-\frac{1}{2V}(Z_{2}-Z_{1}^{2}),$$ (3)

$$C(T)=4B^{2}(T)-\frac{1}{3V}(Z_{3}-3Z_{2}Z_{1}+2Z_{1}^{3}).$$ (4)

### 3.1 Classical and Semiclassical Calculations

The explicit expression for the partition functions and hence the coefficients *Z_k_* appearing in Eqs. (3) and (4) depends on the framework in which the calculations are performed. In classical statistical mechanics (including the correct Boltzmann counting) one has

$$Z_{N}^{\text{class}}=\int d^{3}x_{1}\ldots d^{3}x_{N}\;\exp(-\beta U(x_{1},\ldots ,x_{N})),$$ (5)

where *β* = 1/*k*_B_ *T*, *k*_B_ denotes the Boltzmann constant and *U*(***x***_1_,…, ***x****_N_*) is the total potential energy of a configuration with *N* particles at the positions ***x***_1_,…, ***x****_N_*.

In this case, the third virial coefficient *C*^class^(*T*) is given by the sum of a term depending on the two-body potential *U*_2_(*r*), $C_{2-\text{body}}^{\text{class}}(T)$, and a term depending on the three-body potential, $C_{2-\text{body}}^{\text{class}}(T)$, given by

$$C^{\text{class}}(T)=C_{2-\text{body}}^{\text{class}}(T)+C_{3-\text{body}}^{\text{class}}(T),$$ (6)

$$C_{2-\text{body}}^{\text{class}}(T)=-\frac{8\pi^{2}}{3}\int dr_{12}\;dr_{13}\;d\cos\theta \;r_{12}^{2}\;r_{13}^{2}\;(e^{-\beta U_{2}(r_{12})}-1)\times (e^{-\beta U_{2}(r_{13})}-1)(e^{-\beta U_{2}(r_{23})}-1),$$ (7)

$$C_{3-\text{body}}^{\text{class}}(T)=-\frac{8\pi^{2}}{3}\int dr_{12}\;dr_{13}\;d\;\cos\theta \;r_{12}^{2}\;r_{13}^{2}\;\left[e^{-\beta U_{3}\left(r_{12},r_{13},r_{23}\right)}-1\right]\times e^{-\beta [U_{2}(r_{12})+U_{2}(r_{13})+U_{2}(r_{23})]}.$$ (8)

We have denoted by *θ* the angle between the vectors ***r***_12_ and ***r***_13_. The distance between the particles labeled 2 and 3 is therefore given by

$$r_{23}=\sqrt{r_{12}^{2}+r_{13}^{2}-2r_{12}r_{13}\;\cos\;\theta }.$$ (9)

The classical formulae are accurate enough for heavy particles at high temperature. If this limit is not attained, quantum diffraction effects (Heisenberg uncertainty) become appreciable, as is the case for hydrogen and helium at and below room temperature. As long as the quantum effects can be considered a small correction to the classical behavior, the expressions given above can be corrected by including the first term in the expansion of the full quantum expression in even powers of *ħ*.

The expression for the first quantum correction to the third virial coefficient has been evaluated by Yokota [10]. Setting *a*^2^ = *ħ*^2^*β*/*m*, where *m* is the mass of the particles under consideration, the semiclassical expression for the third virial coefficient turns out to be

$$C^{\text{semi}}(T)=C^{\text{class}}(T)+a^{2}C_{1}(T),$$ (10)

$$C_{1}(T)=8B_{1}(T)B^{\text{class}}(T)-2V\;B_{1}(T)+\frac{\beta^{2}}{36}\int d^{3}r_{12}d^{3}r_{13}\;e^{-\beta U}\left(KUU\right)$$ (11)

where

$$B_{1}(T)=\frac{\beta^{2}}{24}\int d^{3}\;r\;e^{-\beta U_{2}}{\left(\frac{dU_{2}}{dr}\right)}^{2},$$ (12)

$$KUU=\nabla_{12}U\cdot \nabla_{12}U+\nabla_{13}U\cdot \nabla_{13}U+\frac{1}{2}(\nabla_{12}U\cdot \nabla_{13}U+\nabla_{13}U\cdot \nabla_{12}U).$$ (13)

The classical and semiclassical values of *C*(*T*) have been obtained by direct numerical integration of Eqs. (6) and (10). We have used the QAG adaptive algorithm together with the Gauss-Kronrod 15-point rule as implemented in the GNU Scientific Library [27]. The interaction has been neglected beyond a cutoff length of *L* = 4 nm. We have checked that using a larger cutoff value does not appreciably affect the results, and the same cutoff was also used in performing the path-integral Monte Carlo calculations described below.

### 3.2 Path-Integral Monte Carlo

In the framework of non-relativistic quantum statistical mechanics, the expression for the quantity *Z_N_* of Eq. (2) becomes

$$Z_{N}=\Lambda_{m}^{3N}\sum_{k}\sum_{\pi }\langle k|\exp\left(-\beta {\hat{H}}_{N}\right){\hat{P}}_{\pi }|k\rangle ,$$ (14)

where ${\hat{H}}^{N}$ is the *N*-body Hamiltonian operator, |*k*〉 denotes a complete set of *N*-particle states and ${\hat{P}}_{\pi }$ is a permutation operator, with the proper sign to take into account the bosonic or fermionic nature of the particles. $\Lambda_{m}=h/\sqrt{2\pi mk_{B}T}$ denotes the thermal de Broglie wavelength for a particle of mass *m*. In the following, we will not be concerned with temperatures so low that exchange effects play a relevant role [28] and hence we will approximate the sum over ${\hat{P}}_{\pi }$ with the identity operator (Boltzmann statistics).

Equation (14) is the starting point to derive the path-integral expressions for the second and third virial coefficients, using Eqs. (3) and (4). In order to avoid cumbersome notation, we will present the derivation in detail in the case of the second virial coefficient. This will allow us to establish some useful notation that will be used to present in the most compact form possible the path-integral formulae for the third virial coefficient.

#### 3.2.1 Second Virial Coefficient

The path-integral formula for the second virial coefficient of Eq. (3), using the quantum-mechanical expression of Eq. (14) in the case of Boltzmann statistics, is readily obtained by first performing a canonical transformation to the center of mass ***R*** ≡ (***x***_1_ + ***x***_2_)/2 and relative ***r*** ≡ ***r***_1_ ≡ ***x***_2_ − ***x***_1_ coordinates. In the equation for *Z*_2_, the kinetic energy relative to the center-of-mass motion commutes with the kinetic and potential energy of the relative motion and can be integrated out, obtaining a factor $V/\Lambda_{M}^{3}$, where *M* = 2*m*. As a consequence, *B*(*T*) is proportional to the trace of the Hamiltonian describing the relative motion ${\hat{H}}_{r}={\hat{p}}_{r}^{2}/2\mu +{\hat{U}}_{2}(|r|)\equiv \hat{T}+{\hat{U}}_{2}$, where *μ* = *m*/2 is the reduced mass of the pair and ${\hat{p}}_{r}$ is the momentum conjugated to the relative coordinate ***r***.

The resulting expression

$$Z_{2}=\frac{\Lambda_{m}^{6}}{2\Lambda_{M}^{3}}\int d^{3}r\langle r|exp\left(-\beta {\hat{H}}_{r}\right)|r\rangle ,$$ (15)

can then be evaluated by performing a Trotter expansion of the kinetic and potential energies of the relative motion,

$$e^{-\beta (\hat{T}+{\hat{U}}_{2})}=\underset{P\to \infty }{\lim}{\left(e^{-\beta \hat{T}/P}\;e^{-\beta {\hat{U}}_{2}/P}\right)}^{P},$$ (16)

keeping a finite value of the Trotter index *P* and inserting *P* − 1 completeness relations 1 = ∫ d***r****_i_* |***r****_i_*〉 〈***r****_i_*|(*i* = 2,…, *P*) between each of the *P* factors in Eq. (16). The matrix elements of the kinetic energy operator can be evaluated explicitly, obtaining

$$\langle r_{i}|\exp\left(-\beta \frac{{\hat{p}}^{2}}{2\mu P}\right)|r_{j}\rangle =\frac{P^{3/2}}{\Lambda_{\mu }^{3}}\exp\left(-\frac{K}{2}{\left(r_{i}-r_{j}\right)}^{2}\right),$$ (17)

where $K=2\pi P/\Lambda_{\mu }^{2}$. The final outcome of this chain of equivalences is to map the calculation of the quantum partition function of Eq. (15) to the calculation of the classical partition function of ring polymers with *P* beads each [29]. The resulting expression can be simplified by introducing the coordinates ***r*** = ***r***_1_, Δ***r****_i_* = ***r****_i_*_+1_ − ***r****_i_*(*i* = 1,…, *P* − 1) and letting

$${\bar{U}}_{2}(r)=\frac{1}{P}\sum_{i=1}^{P}U_{2}(r_{i}),$$ (18)

indicate the average of the two-body potential over the positions occupied by the *P* beads of a given ring polymer. Performing analogous manipulations in the rather trivial case of *Z*^2^_1_ produces the following expression for the second virial coefficient:

$$B(T)=-\frac{1}{2}\int d^{3}r\prod_{i=1}^{P-1}d^{3}\Delta r_{i}\left(e^{-\beta {\bar{U}}_{2}(r)}-1\right)\times F_{\text{ring}}(\mu ;\Delta r_{1},\ldots ,\Delta r_{P-1}),$$ (19)

where *F*_ring_ is the probability of finding a ring polymer configuration in the ideal gas phase, as shown in Ref. [30], and is given by

$$F_{\text{ring}}(\mu ;\Delta r_{1},\ldots ,\Delta r_{P-1})=\Lambda_{\mu }^{3}{\left(\frac{P^{3/2}}{\Lambda_{\mu }^{3}}\right)}^{P}\times \exp\left[-\frac{K}{2}\sum_{i=1}^{P}\Delta r_{i}^{2}\right],$$ (20)

where Δ***r****_P_* = ***r****_P_* − ***r***_1_.

The second virial coefficient can also be written as

$$\begin{array}{l} B(T)=-\frac{1}{2}\int d^{3}r(e^{-\beta V_{2,\text{eff}}(r)}-1)\\ =-2\pi \int dr\;r^{2}(e^{-\beta V_{2,\text{eff}}(r)}-1), \end{array}$$ (21)

where the effective potential is defined as

$$e^{{}^{-\beta V_{2,\text{eff}}(r)}}=\int \prod_{i=1}^{P-1}d^{3}\Delta r_{i}\;e^{{}^{{}^{-\beta {\bar{U}}_{2}(r)}}}\times F_{\text{ring}}(\mu ;\Delta r_{1},\ldots ,\Delta r_{P-1}),$$ (22)

that is, by averaging the factor exp (−*βŪ*_2_) over ring polymer configurations drawn from an ideal gas distribution and having one bead at the point ***r***. In the classical limit *ħ* → 0, the ring polymers shrink to a point, and hence Eq. (21) recovers the classical result.

### 3.2.2 Third Virial Coefficient

The same reasoning that was followed to derive the path-integral expression for the second virial coefficient *B*(*T*) in Eq. (19) can be applied to the case of the third virial coefficient. In this case it is useful to evaluate the expectation values over three-body operators after having performed a canonical transformation to the Jacobi coordinates ***R***, ***r***, ***ρ*** and the corresponding momenta ***P***, ***p***, ***π***, defined as:

$$R=\frac{1}{3}(x_{1}+x_{2}+x_{3}+)\;;\;P=p_{1}+p_{2}+p_{3}$$ (23)

$$r=x_{2}-x_{1}\;;\;p=\frac{1}{2}(p_{2}-p_{1})$$ (24)

$$\rho =x_{3}-\frac{1}{2}(x_{1}+x_{2})\;;\;\pi =\frac{1}{3}(2p_{3}-p_{1}-p_{2}).$$ (25)

As in the case of *B*(*T*) the center-of-mass motion can be integrated out, but the Trotter factorization introduces two different ring polymers, corresponding to the coordinates ***r*** and ***ρ*** ; we note in passing that the masses associated with these degrees of freedom are *M_r_* = *m*/2 and *M_ρ_* = 2*m*/3, respectively, and that the total potential energy of a three-body configuration,

$$U(x_{1},x_{2},x_{3})\equiv U_{3}(|x_{2}-x_{1}|,|x_{3}-x_{2}|,|x_{3}-x_{1}|)+\sum_{i<j=1}^{3}U_{2}(|x_{i}-x_{j}|),$$ (26)

is a function of the coordinates ***r*** and ***ρ*** only. As a final result, the expression for *C*(*T*) can be written as

$$C(T)=4B^{2}(T)-\frac{1}{3}\int d^{3}\;r\;d^{3}\rho \prod_{i=1}^{P-1}d^{3}\Delta r_{i}\prod_{i=1}^{P-1}d^{3}\Delta \rho_{i}\times \left(e^{-\beta \bar{U}(r;\rho )}-e^{-\beta {\bar{U}}_{2}(r)}-e^{-\beta {\bar{U}}_{2}(r+\rho /2)}-e^{-\beta {\bar{U}}_{2}(r-\rho /2)}+2\right)\times F_{\text{ring}}(M_{r};\Delta r_{1},\ldots ,\Delta r_{P-1})F_{\text{ring}}(M_{\rho };\Delta \rho_{1},\ldots ,\Delta \rho_{P-1}),$$ (27)

where we have defined, analogously to Eq. (18),

$$\bar{U}(r;\rho )=\frac{1}{P}\sum_{i=1}^{P}U(r_{i};\rho_{i}),$$ (28)

as the average of the potential energy of the three bodies over the positions indicated by the beads of the two given ring polymers corresponding to the Jacobi coordinates ***r*** = ***r***_1_ and *ρ* = *ρ*_1_. The three exponentials of *Ū*_2_ appearing in Eq. (27) come from the three terms $Z_{2}Z_{1}^{2}$ of Eq. (4), when the two-body integral is written as a function of the coordinates

$$r_{21}=x_{2}-x_{1}=r,$$ (29)

$$r_{31}=x_{3}-x_{1}=\rho +\frac{r}{2},$$ (30)

$$r_{32}=x_{3}-x_{2}=\rho -\frac{r}{2},$$ (31)

respectively.

The integrals over the variables Δ***r****_i_* and Δ***ρ****_i_* allow one to define effective two- and three-body potentials as averages over the two kinds of ring polymers, analogously to Eq. (22)

$$e^{-\beta U_{e\text{ff}}(r,\rho )}=\int \prod_{i=1}^{P-1}d^{3}\Delta r_{i}\prod_{i=1}^{P-1}d^{3}\Delta \rho_{i}\;e^{-\beta \bar{U}(r;\rho )}\times F_{\text{ring}}(M_{r};\Delta r_{1},\ldots ,\Delta r_{P-1})F_{\text{ring}}(M_{\rho };\Delta \rho_{1},\ldots ,\Delta \rho_{P-1}),$$ (32)

$$e^{-\beta U_{2,e\text{ff}}(|x|)}=\int \prod_{i=1}^{P-1}d^{3}\Delta r_{i}\prod_{i=1}^{P-1}d^{3}\Delta \rho_{i}\;e^{-\beta {\bar{U}}_{2}(|x|)}\times F_{\text{ring}}(M_{r};\Delta r_{1},\ldots ,\Delta r_{P-1})F_{\text{ring}}(M_{\rho };\Delta \rho_{1},\ldots ,\Delta \rho_{P-1}),$$ (33)

where ***x*** can be either ***r*** or ***ρ*** + ***r***/2 or ***ρ*** − ***r***/2. The final expression for *C*(*T*) is hence

$$C(T)=4B^{2}(T)-\frac{1}{3}\int d^{3}\;r\;d^{3}\;\rho \times \left(e^{-\beta U_{\text{eff}}(r;\rho )}-e^{-\beta U_{2,\text{eff}}(r)}-e^{-\beta U_{2,\text{eff}}(r+\rho /2)}-e^{-\beta U_{2,\text{eff}}(r-\rho /2)}+2\right).$$ (34)

Given the rotational symmetry of the system, the volume of integration can be written as

$$d^{3}rd^{3}\rho =4\pi r^{2}dr\;2\pi \rho^{2}d\rho \;d\;\cos\;\Theta \;,$$ (35)

where *r* and *ρ* are the moduli of the vectors ***r*** and ***ρ***, respectively, while *Θ* is the angle between them.

In the actual calculation, it is useful to write the square of the second virial coefficient as

$$B^{2}(T)=\frac{1}{4}\int d^{3}\;r\;d^{3}\;\rho \left(e^{-\beta U_{2,\text{eff}}(r)}-1\right)\left(e^{-\beta U_{2,\text{eff}}(\rho )}-1\right),$$ (36)

so that the difference of two integrals in Eq. (34) can be evaluated as the integral of the difference. Note that in Eq. (36) the coordinate ***ρ*** is associated with a particle having mass *M_r_* and not *M_ρ_* as in Eqs. (32) and (33).

The calculation of the third virial coefficient in the path-integral formalism follows directly from Eq. (34), which shows that *C*(*T*) is given as a three-dimensional integral using effective two- and three-body potentials, given by Eqs. (33) and (32), respectively. Since for each of the atomic positions the effective potentials are obtained as a costly average over configurations of ring polymers, we chose to evaluate Eq. (34) using a Monte Carlo integration procedure, namely the VEGAS algorithm [27, 31, 32]. We used *N* = 5 × 10^5^ integration points for the production stage of the algorithm and half as many for the equilibration stage.

For each of the atomic configurations considered in the course of the Monte Carlo integration, we generate *n* = 200 ring polymers for the ***r*** and ***ρ*** coordinates, distributed according to the probability *F*_ring_. This can be done very efficiently using an interpolation formula due to Levy [18, 33]. For each of the ring polymers, we evaluate the corresponding average potentials *Ū*_2_ and *Ū* and accumulate their Boltzmann factors to calculate the effective potentials for the given configuration according to Eqs. (32) and (33).

In order to estimate the statistical uncertainty of the values of the third virial coefficient so obtained, we perform 16 of these calculations for each value of *T*, starting with different seeds for the random number generator.

The final value for *C*(*T*) is obtained as the average of the values coming from the 16 independent calculations, and its Type A uncertainty is estimated as the standard error of the mean from the same set of values. Notice that this uncertainty takes into account the statistical error resulting from use of both a finite *N* and a finite *n* in the calculations.

Finally, let us discuss the choice of the Trotter index *P*. Since the path-integral method is exact in the limit of large *P*, we fixed this number by calculating *B*(*T*) with our method for progressively increasing values of the Trotter index *P*, until our results matched those performed with the phase-shift method [6]. We found that the choice *P* = 7 + 2400 K/*T* was enough to reach convergence in a wide range of temperatures, and we similarly checked that the same value of *P* was sufficient in the case of *C*(*T*) at 273.16 K. The offset of 7 in the expression for *P* is due to the approximation inherent in Levy’s interpolation formula [18, 33].

## 4. Results

### 4.1 Third Virial Coefficients

We calculated *C*(*T*) as described in Sec. 3.2 for the potential-energy surface obtained by combining the *ϕ*_07_ pair potential referenced in Sec. 2.1 with the three-body potential (CC) referenced in Sec. 2.2. Calculations were performed over a wide range of temperatures, as shown in Table 1. In addition to round values, some temperatures were chosen due to their importance in metrology. For example, our lowest temperature of 24.5561 K is the value assigned to the triple point of neon in the International Temperature Scale of 1990 (ITS-90) [34]. It should be noted that *T* in our calculations is the thermodynamic temperature, which may differ on the order of 0.005 % from the corresponding ITS-90 temperature [3]. We included temperatures up to 10 000 K, in order to match the range covered by Hurly and Mehl [6] for *B*(*T*). Temperatures below those shown in Table 1 were not achievable with available computing resources (the calculations for 24.5561 K took approximately 700 hours of CPU time with 2.2 GHz processors for each combination of two- and three-body potentials.)

### 4.2 Uncertainty Analysis

The largest contribution to the uncertainty of *C*(*T*) comes from imperfect knowledge of the three-body part of the potential energy, but there are smaller contributions that must also be considered. The following three uncertainty contributions are identified:

- Uncertainty in the pair potential. The pair potential *ϕ*_07_ (see Sec. 2.1) used for our calculations has an unknown systematic error (difference from the true pair potential) that will produce a corresponding uncertainty in *C*(*T*). In Ref. [6], it is stated that the pair potentials $\phi_{07}^{-}$ and $\phi_{07}^{+}$ provide lower and upper bounds, respectively, to the true potential at a level of confidence corresponding to an expanded uncertainty at the *k* = 2 level. *C*(*T*) calculated from these perturbed potentials will represent an uncertainty at the same *k* = 2 level. Therefore, we take as the standard uncertainty (*k* = 1) from this source one-fourth of the difference between *C*(*T*) calculated with $\phi_{07}^{+}$ and with $\phi_{07}^{-}$
- Uncertainty in the three-body potential. As explained in Sec. 2.2, we consider the CC+ and CC− potentials to bound the true three-body potential at a level of confidence corresponding to an expanded uncertainty at the *k* = 2 level. Therefore, we take as the standard uncertainty (*k* = 1) from this source one-fourth of the difference between *C*(*T*) calculated with CC+ and with CC−. At all temperatures studied, this is the largest of the three uncertainty contributions.
- Uncertainty in the convergence of the PIMC calculation. This is estimated as the standard deviation of the mean from 16 independent Monte Carlo samples, as described near the end of Sec. 3.2.2.

The first two of these contributions are systematic (Type B) errors, while the third is strictly statistical (Type A). The three contributions are combined in quadrature, and the resulting standard uncertainty is multiplied by a coverage factor *k* = 2 to produce the expanded uncertainty *U*(*C*) in Table 1.

For purposes of illustration, we review the uncertainty calculation (sometimes keeping insignificant digits for clarity) for the point at 273.16 K. The difference between *C* calculated with the $\phi_{07}^{+}$ and $\phi_{07}^{-}$ potentials is 0.062 cm^6^ · mol^−2^, so this component of the uncertainty is 0.0155 cm^6^ · mol^−2^. The difference between calculations with the CC+ and CC− three-body potentials is 0.665 cm^6^ · mol^−2^, so this component of the standard uncertainty is 0.166 cm^6^ · mol^−2^. The PIMC integration with the CC three-body potential yields *C* = 112.73 cm^6^ · mol^−2^ with integration uncertainty of 0.03 cm^6^ · mol^−2^. Combining the three contributions in quadrature yields *u*_c_ (*C*) = 0.1696 cm^6^ · mol^−2^, which when multiplied by two yields an expanded uncertainty with coverage factor *k* = 2 of *U*(*C*) = 0.339 cm^6^ · mol^−2^.

### 4.3 Correlation for Results

For convenience in interpolation, we have correlated the results for *C*(*T*) in Table 1 as a function of absolute temperature *T* with a simple empirical expression:

$$\frac{C(T)}{{\text{cm}}^{6}\cdot {\text{mol}}^{-2}}=\sum_{i=1}^{6}a_{i}{\left(T*\right)}^{b_{i}}\;,$$ (37)

where T* = *T*/(100 K) and the parameters *a_i_* and *b_i_* are given in Table 2. Equation (37) reproduces all the *C*(*T*) values in Table 1 to within better than 0.11 %, which is smaller than the standard uncertainty *u*_c_(*C*) and similar to the portion of the uncertainty due to Type A sources of error. For temperatures above 2000 K, where our calculated values of *C*(*T*) are sparse, a finer grid of semiclassical calculations (which effectively coincide with the PIMC calculations at these temperatures; see Sec. 4.5) was used to guide the interpolation.

Equation (37) may be easily differentiated to obtain $\frac{dC}{dT}$ and $\frac{d^{2}C}{dT^{2}}$, which are useful in the context of acoustic measurements. We know of no rigorous way to estimate the uncertainty of these derivatives, but some idea of their quality may be obtained from the uncertainty in *C*(*T*) and the knowledge that the majority of that uncertainty comes from Type B (systematic) sources that would mostly cancel out in the computation of derivatives.

It is important to note that Eq. (37) is strictly for interpolation. It is only valid for the range of temperatures covered in Table 1 (24.5561 K to 10 000 K). The behavior of Eq. (37) outside this range is physically reasonable for short distances, but for example it does not reproduce the maximum in *C*(*T*) that is believed to exist near 4 K [35].

### 4.4 Comparison With Experiment

In this section, we compare our results with selected experimental data. This is not a comprehensive review of experimental work; the purpose of the comparisons is simply to show the scatter of the existing data and to demonstrate the improvement in uncertainty of *C*(*T*) obtained in our calculations. Therefore, we have selected for comparison the most recent data sets and those with well-documented uncertainties, adding other data sets in a few cases in order to cover the entire temperature range of the experimental data.

**Fig. 1:**
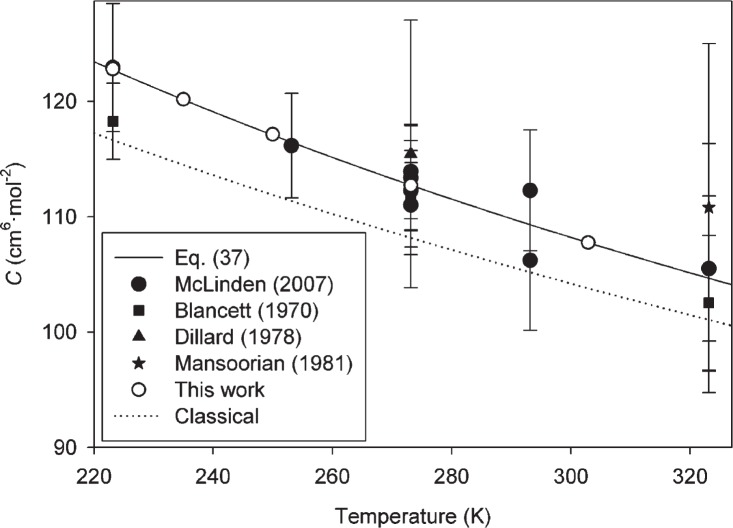
Comparison of *C*(*T*) calculated in this work with experimental values at near-ambient temperatures. Error bars on experimental points represent expanded uncertainties with coverage factor *k* = 2; expanded uncertainties for this work (given in Table 1) are not shown on the figure because the error bars would be similar in size to the symbols.

We begin with the near-ambient temperature range, which is of the most interest for metrology and has been the subject of the most experimental study. Figure 1 compares our results with four sources of experimental data [36–39]. The error bars for the experimental points in this and subsequent figures represent an expanded uncertainty with coverage factor *k* = 2, corresponding approximately to a 95 % confidence interval. The appropriate comparable uncertainty corresponding to our calculated results is the last column of Table 1; these uncertainties are not shown on Fig. 1 because they are approximately the size of the symbols themselves.

**Fig. 2:**
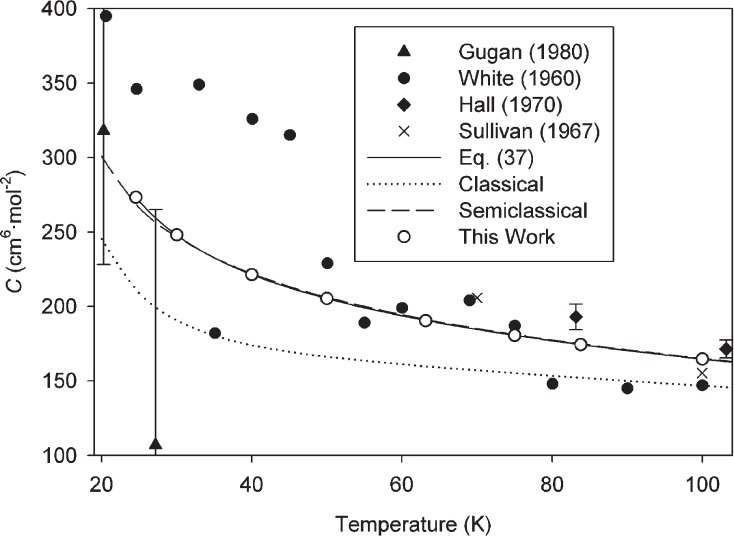
Comparison of *C*(*T*) calculated in this work with experimental values at low temperatures. Error bars on experimental points (drawn where reported) represent expanded uncertainties with coverage factor *k* = 2; expanded uncertainties for this work (given in Table 1) are not shown on the figure because the error bars would be smaller than the symbols.

**Fig. 3:**
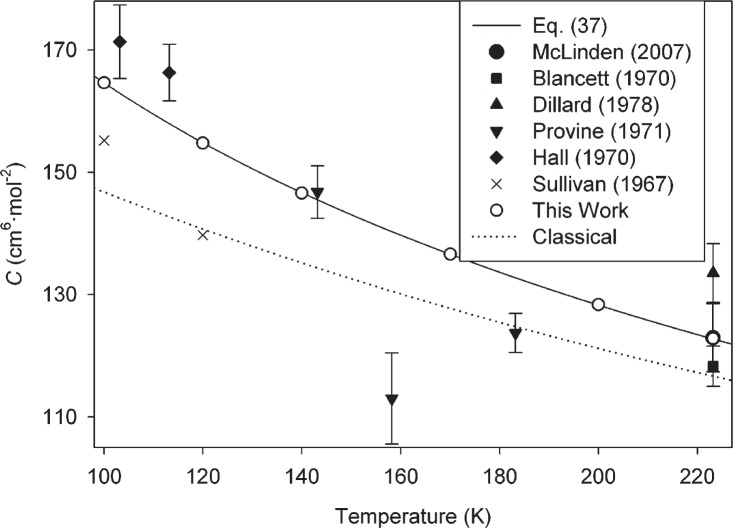
Comparison of *C*(*T*) calculated in this work with experimental values at moderately low temperatures. Error bars on experimental points (drawn where reported) represent expanded uncertainties with coverage factor *k* = 2; expanded uncertainties for this work (given in Table 1) are not shown on the figure because the error bars would be smaller than the symbols.

**Fig. 4:**
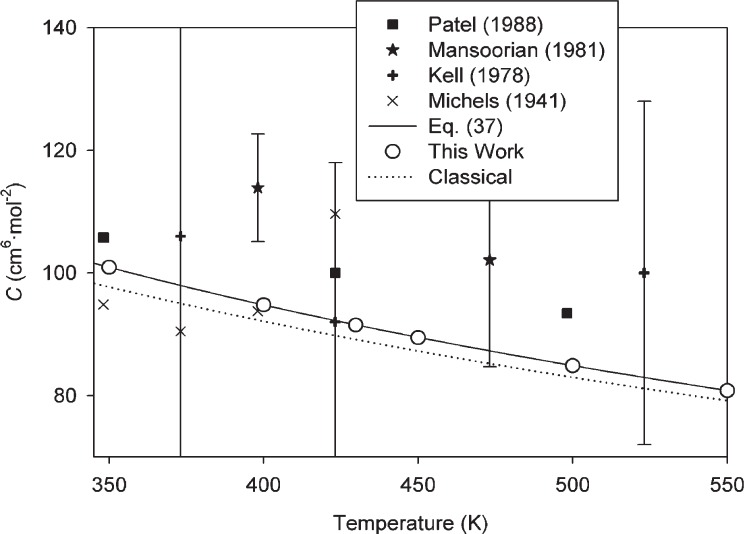
Comparison of *C*(*T*) calculated in this work with experimental values at high temperatures. Error bars on experimental points (drawn where reported) represent expanded uncertainties with coverage factor *k* = 2; expanded uncertainties for this work (given in Table 1) are not shown on the figure because the error bars would be smaller than the symbols.

These sources of experimental data are fairly consistent; in particular the data of Blancett *et al.* [36] and of McLinden and Lösch-Will [39] have found use in metrology because their expanded uncertainties of approximately 3 cm^6^ · mol^−2^ to 5 cm^6^ · mol^−2^ have been the best available. Our results are fully consistent with these measurements, but have an uncertainty smaller by approximately one order of magnitude (for example, our *U*(*C*) at 273.16 K is 0.34 cm^6^ · mol^−2^). Values of *C*(*T*) calculated classically are also shown in Fig. 1. Semiclassical results are not shown; they lie slightly above the full PIMC results but not enough to be clearly visible on the scale of the figure. Figures 2–4 show similar comparisons with selected data at low temperatures [40–43], moderately low temperatures [36, 37, 39, 41, 42, 44], and high temperatures [38, 45–47], respectively. In cases where experimental points are shown without error bars, the original paper did not report uncertainties. In all three figures, the uncertainty of the present calculations (see Table 1) is smaller than the size of the symbols. Classical results are shown on all three figures; only on the low-temperature Fig. 2 are the semiclassical results distinguishable from the PIMC calculations on the scale of the figure. For all these temperature ranges, our results are generally consistent with the available data. Given the scatter and uncertainties of the experimental data, it is reasonable to say that our calculations reduce the uncertainty in these temperature ranges by more than an order of magnitude. There appear to be no experimental data for *C*(*T*) available for helium at temperatures higher than those shown in Fig. 4. While estimates for these higher temperatures can be made based on extrapolation, Lennard-Jones potentials, etc., undoubtedly the present results are more reliable than any such estimates.

After this work was completed, Gaiser and Fellmuth [48] published new experimental data for *C*(*T*) of helium from 3.7 K up to 26 K. Only our lowest-temperature point (24.5561 K) overlaps with their data. Interpolating their results to this temperature produces a value of approximately 281 cm^6^· mol^−2^ with standard uncertainty *u* (*C*) of approximately 11 cm^6^· mol^−2^. This agrees within its uncertainty with our result given in Table 1.

### 4.5 Accuracy of Classical and Semiclassical *C*(*T*)

Because of the high computational demands of the PIMC calculation, it is natural to consider whether, at sufficiently high temperatures, adequate results may be obtained from the simpler semiclassical calculation or even the much simpler classical calculation. One might consider such a calculation “adequate” if it differed from the full PIMC calculation by less than the expanded uncertainty *U*(*C*) in Table 1.

The classical calculation significantly underestimates *C*(*T*) even at quite high temperatures. For example, at 1000 K it differs from the PIMC result by more than 0.6 cm^6^ · mol^−2^, more than twice the expanded uncertainty of the PIMC result. Only above approximately 2000 K is the classical calculation adequate in the sense defined above. The degree to which the classical calculation is in error at lower temperatures can be seen in Figs. 1–4. While this error is comparable to the scatter of the experimental data in many cases, it is much larger than the uncertainty in our calculations.

**Fig. 5:**
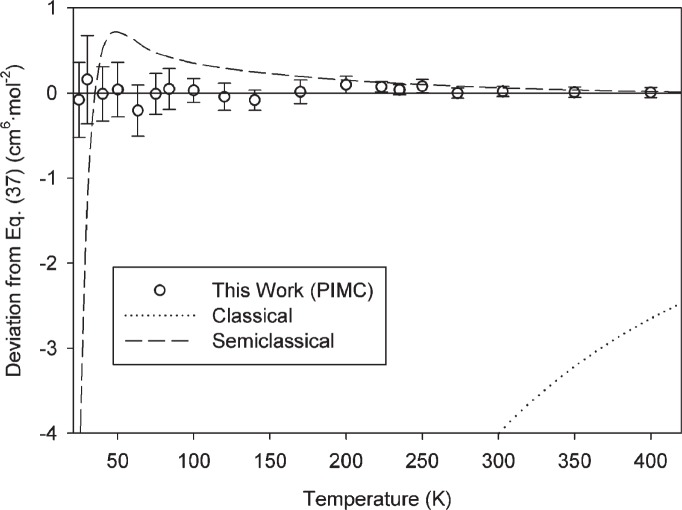
Comparison of classical and semiclassical values of *C*(*T*) to PIMC values as represented by Eq. (37). Error bars on PIMC points in this figure represent only the Type A uncertainty at a *k* = 2 level.

The semiclassical calculation is significantly better, closely reproducing the full PIMC calculations down to about 200 K and producing adequate results at temperatures as low as 120 K. In Fig. 5, we plot the deviation of the semiclassical calculation from Eq. (37) as a function of temperature. Figure 5 also shows the classical calculations and the individual PIMC calculations. Because systematic errors from the potential contribute similarly to both PIMC and semiclassical calculations, the error bars in Fig. 5 represent (at a *k* = 2 level) only the Type A uncertainty due to the convergence of the PIMC calculations.

### 4.6 Contribution of Three-Body Potential to *C*(*T*)

We can examine the contribution of the three-body potential to *C*(*T*). While this is the most important factor in the overall uncertainty (see Sec. 4.2). it actually contributes only a small fraction of the value of *C*(*T*) itself, on the order of 1 % or 2 %

Interestingly, the sign of the three-body contribution changes; it contributes positively to *C*(*T*) at low temperatures and negatively at moderate and high temperatures. This reflects the fact that dispersion forces (for which the net three-body contribution to the energy is generally positive) are more important at low temperatures, while exchange repulsion forces (for which the net three-body effect is generally negative) are more important at high temperatures. The “crossover” where the net three-body contribution to *C*(*T*) is zero occurs at approximately 170 K.

## 5. Discussion

We have computed *C*(*T*) for helium from state-of-the-art pair and three-body potentials, producing values with uncertainties smaller than those from experiment by at least an order of magnitude. For temperatures at and above the neon triple point, these values (as represented by Eq. (37)) provide a significant improvement for this useful quantity in metrology.

The largest source of uncertainty in our calculations of *C*(*T*) is the uncertainty in the three-body potential; therefore, this provides the greatest opportunity for improvement. As mentioned in Sec. 2.2, some very recent work [25], completed after our calculations were finished, has analyzed the three-body potential at the full-configuration-interaction (FCI) level of theory. This FCI potential is significantly more accurate than the CC and SAPT potentials developed earlier in Ref. [23]. Given sufficient computing resources (and characterization of the uncertainty of the FCI three-body potential), our calculations here could be repeated with the FCI potential and smaller uncertainties obtained. This is planned for future work.

To give some indication of the improvement expected, in Table 3 we show a few values of *C*_FCI_ calculated with the new three-body potential of Ref. [25]. We are not yet in a position to assign uncertainties to *C*_FCI_, but our preliminary estimate is that the uncertainties will be reduced by a factor of approximately three compared to our current values. It is clear from Table 3 that the values of *C*_FCI_ are fully consistent with the values of *C*(*T*) computed in this work (so the uncertainty assigned here to the CC three-body potential of Ref. [23] was reasonable), but that our values are systematically lower by a small amount. Table 3 also shows values *C*_SAPT_ calculated with the SAPT three-body potential of Ref. [23]. These values are much further away from the accurate FCI results than those from the CC potential, demonstrating at the level of *C*(*T*) the superiority of the CC three-body potential, which the authors of Ref. [25] observed at the level of the potential itself. This provides further justification for our use of only the CC potential from Ref. [23] in our calculation of *C*(*T*).

Work is also underway to reduce the uncertainty in the pair potential [49]. While improving the pair potential will significantly reduce the uncertainty in the pair quantities such as *B*(*T*) calculated by Hurly and Mehl [6], it will not be as helpful for *C*(*T*) where the pair potential contributes a relatively small portion of the overall uncertainty. When an improved pair potential (including characterization of its uncertainty) is completed, that would be an opportune time to recalculate *C*(*T*) with the FCI three-body potential of Ref. [25] in order to provide a complete and consistent set of state-of-the-art *ab initio* property values for helium.

It would, of course, be desirable to extend this work to lower temperatures. However, the required computing time per point scales approximately as 1/*T*, and the computation at 24.5561 K already took over 4000 CPU-hours. Extending the range much below 20 K would require an improvement in the technique or greatly increased computing power. At very low temperatures, around 7 K and below, [28] the use of Boltzmann statistics will begin to be in error. Extension of the PIMC method to incorporate the correct statistics for ^4^He (a boson) is feasible but would introduce additional complexity and computing time.

There would also be some interest in *C*(*T*) for the isotope ^3^He. Calculating *C*(*T*) for ^3^He would be a straightforward extension of the current work, although the smaller mass (and therefore larger quantum effects) would somewhat increase the computational requirements. Unfortunately, the greatest interest in ^3^He in metrology is for thermometry at very low temperatures that are currently beyond our reach with this method.

For metrology at moderately high pressures, the fourth virial coefficient *D*(*T*) might also be of interest. The potential energy should be adequately described by the sum of pair and three-body potentials; the relatively small size of the three-body contribution compared to the pair contribution suggests that nonadditivity of the potential at the four-body level should be tiny (this could be checked by *ab initio* calculations at selected configurations). PIMC calculation of *D*(*T*) would be computationally prohibitive, except perhaps at quite high temperatures where quantum effects are small. However, as discussed in Sec. 4.5, semiclassical calculations are accurate for *C*(*T*) above approximately 200 K; it is reasonable to assume that this would also be true for *D*(*T*). In the paper deriving the semiclassical perturbation approach to *C*(*T*), Yokota [10] indicates that extension of the approach to *D*(*T*) is feasible, but to our knowledge it has not been done. Such an extension of the semiclassical approach could provide highly accurate values of *D*(*T*) for helium in the important metrological range near room temperature.

Values of $\frac{dC}{dT}$ and $\frac{d^{2}C}{dT^{2}}$ are of interest for acoustic measurements, where they are related to the acoustic virial coefficients. These may be obtained by differentiating the interpolating Eq. (37), but the uncertainty in such derived values is difficult to quantify. Direct calculation of $\frac{dC}{dT}$ and, possibly, $\frac{d^{2}C}{dT^{2}}$ might be feasible through the use of histogram reweighting together with the technique of thermodynamic integration to obtain *N*-particle functions and, as a consequence, the virial coefficients from Eqs. (3) and (4). Evaluation of the partition functions might also provide an alternative route for the calculation of *D*(*T*) and/or the incorporation of quantum statistics (bosonic or fermionic) at low temperatures.

## Figures and Tables

**Table 1:** Third virial coefficients *C*(*T*) calculated in this work and our estimates (see Sec. 4.2) of their expanded (*k* = 2) uncertainties *U*(*C*)

*T* (K)	*C* (cm^6^ · mol^−2^)	*U*(*C*) (cm^6^ · mol^−2^)
24.5561	273.34	1.26
30.0	248.08	1.54
40.0	221.36	0.96
50.0	205.31	0.78
63.15	190.38	0.83
75.0	180.56	0.64
83.806	174.37	0.63
100.0	164.65	0.55
120.0	154.78	0.47
140.0	146.59	0.45
170.0	136.58	0.39
200.0	128.34	0.33
223.152	122.80	0.33
235.0	120.18	0.37
250.0	117.15	0.29
273.16	112.73	0.34
302.915	107.75	0.31
350.0	100.91	0.30
400.0	94.76	0.31
429.75	91.48	0.27
450.0	89.43	0.26
500.0	84.87	0.26
550.0	80.81	0.25
600.0	77.18	0.24
650.0	73.96	0.26
700.0	71.00	0.23
750.0	68.33	0.24
800.0	65.92	0.23
900.0	61.61	0.24
1000.0	57.87	0.24
1200.0	51.80	0.22
1400.0	46.98	0.23
1600.0	43.08	0.22
1800.0	39.81	0.22
2000.0	37.03	0.21
2500.0	31.59	0.21
5000.0	18.31	0.19
10000.0	9.67	0.16

**Table 2:** Coefficients for Eq. (37) for the third virial coefficient of helium

*i*	*a_i_*	*b_i_*
1	−13 337.07	−0.77
2	36 155.73	−0.85
3	−50 678.58	−1.00
4	50 673.92	−1.15
5	−23 876.30	−1.25
6	1 226.921	−1.50

**Table 3:** Selected values of third virial coefficients *C*(*T*) calculated in this work from the CC three-body potential [23] and their expanded (*k* = 2) uncertainties *U*(*C*), along with values *C*_SAPT_ calculated from the SAPT three-body potential [23] and *C*_FCI_ calculated from the new three-body potential of Cencek *et al*. [25]

*T* (K)	*C* (cm^6^ · mol^−2^)	*U*(*C*) (cm^6^ · mol^−2^)	*C*_SAPT_ (cm^6^ · mol^−2^)	*C*_FCI_ (cm^6^ · mol^−2^)
100.0	164.65	0.55	163.98	164.98
200.0	128.34	0.33	127.89	128.60
273.16	112.73	0.34	112.34	112.92
400.0	94.76	0.31	94.41	94.88
1000.0	57.87	0.24	57.54	57.97
